# Single Cell Electrical Characterization Techniques

**DOI:** 10.3390/ijms160612686

**Published:** 2015-06-04

**Authors:** Muhammad Asraf Mansor, Mohd Ridzuan Ahmad

**Affiliations:** 1 Faculty of Biosciences and Medical Engineering, Universiti Teknologi Malaysia, 81310-UTM Skudai, Johor, Malaysia; E-Mail: asraf@biomedical.utm.my; 2 Faculty of Electrical Engineering, Universiti Teknologi Malaysia, 81310-UTM Skudai, Johor, Malaysia

**Keywords:** conversional patch clamp, electrical properties, electrorotation, impedance flow cytometry, microelectrical impedance spectroscopy (µEIS), single cell analysis (SCA)

## Abstract

Electrical properties of living cells have been proven to play significant roles in understanding of various biological activities including disease progression both at the cellular and molecular levels. Since two decades ago, many researchers have developed tools to analyze the cell’s electrical states especially in single cell analysis (SCA). In depth analysis and more fully described activities of cell differentiation and cancer can only be accomplished with single cell analysis. This growing interest was supported by the emergence of various microfluidic techniques to fulfill high precisions screening, reduced equipment cost and low analysis time for characterization of the single cell’s electrical properties, as compared to classical bulky technique. This paper presents a historical review of single cell electrical properties analysis development from classical techniques to recent advances in microfluidic techniques. Technical details of the different microfluidic techniques are highlighted, and the advantages and limitations of various microfluidic devices are discussed.

## 1. Introduction

Study of the cell has emerged as a distinct new field, and acknowledged to be one of the fundamental building blocks of life. Moreover, the cells have unique biophysical and biochemical properties to maintain and sense the physiological surrounding environment to fulfill its specific functions [1,2]. Cellular biophysical properties analysis, such as the electrical, mechanical, optical and thermal characterization of cells, provides critical knowledge to diagnostics, clinical science and pharmaceutical industry [1]. Biophysical properties of cells provide early signals of disease or abnormal condition to the human body, which make it them valuable as potential markers for identifying cancers [3,4,5,6,7], bacteria [8,9,10], toxin detection [11] and the status of tissues [12,13]. Furthermore rapid growing technologies (e.g., conventional patch-clamp, dual nanoprobe-ESEM (environmental scanning electron microscope) and microfluidics) to investigate the biophysical properties of cells have been invented and developed by the researchers in the last decades. The technologies are continually improved make substantial contributions to biology and the clinical research community [14,15].

Single cell analysis (SCA) has become a trend and major topic to engineers and scientists in the last 20 years to develop the experimental tools and technologies able to carry out single cell measurement. In addition, in depth analysis and more fully described activities of cell differentiation and cancer can only be accomplished with single cell analysis [16]. In conventional methods of cellular analysis, population based studies have been utilized for cellular processes such as metabolism, motility, cell growth and proliferation. Population methods use averages of cell properties to measure and predict the biophysical and biochemical parameters of cell. However, this method suffers from inaccurate measurements and often overlooks the essential information available in the cell due to the heterogeneity of cells (e.g., specific gene expression levels) [17]. For this reason, single cell studies have been emphasized to provide biologists and scientists to peer into the molecular machinery of individual cells. Single cell analysis has also been essential to our understanding of some fundamental questions, such as what makes single cells different biophysically, biochemically and functionally. Single cell analysis has been a key in probing of cancer [4,18], and thus helps doctors to develop a prognosis and design a treatment plan for particular patients.

Electrical properties of cells provide some insight and vital information to aid the understanding of complex physiological states of the cell. Cells that experience abnormalities or are infected by bacteria may have altered ion channel activity [19], cytoplasm conductivity and resistance [20,21] and deformability [22]. For instance, red blood cells (RBCs) infected by Plasmodium falciparum, which cause malaria in humans, reduce deformability of the RBC by producing cytoadherence-related neoantigens that increase the rigidity and internal viscosity of the cytomembrane [23,24]. Each RBC which experiences the deformation process, has difference resistance, where the average resistance value of normal RBCs and rigidified RBCs are 14.2 and 19.6 Ω respectively [23]. Since electrical properties of cells have several advantages in cells analysis such as counting, separating, trapping and characterizing of single cell, development of suitable devices for single cell electrical analysis in term of accuracy prediction, portable, and user friendly are very important. In this review, we present an overview of classical technique and microfluidics technique in single cell electrical properties analysis.

## 2. Classical Platforms

The classical technique for a cell’s electrical properties analysis was originated in 1791, when Luigi Galvani conducted the first experiment for measuring electrical activity in animals, which is evoking muscular contractions in frog nerve muscle preparations by electrical stimulation with metal wires [25]. From that study, tools for analyzing a cell’s electrical properties have development over the years. Conventional patch clamp and probing were have been the classical platform tools for characterization of single cell electrical properties.

### 2.1. Conventional Patch Clamp

**Figure 1:**
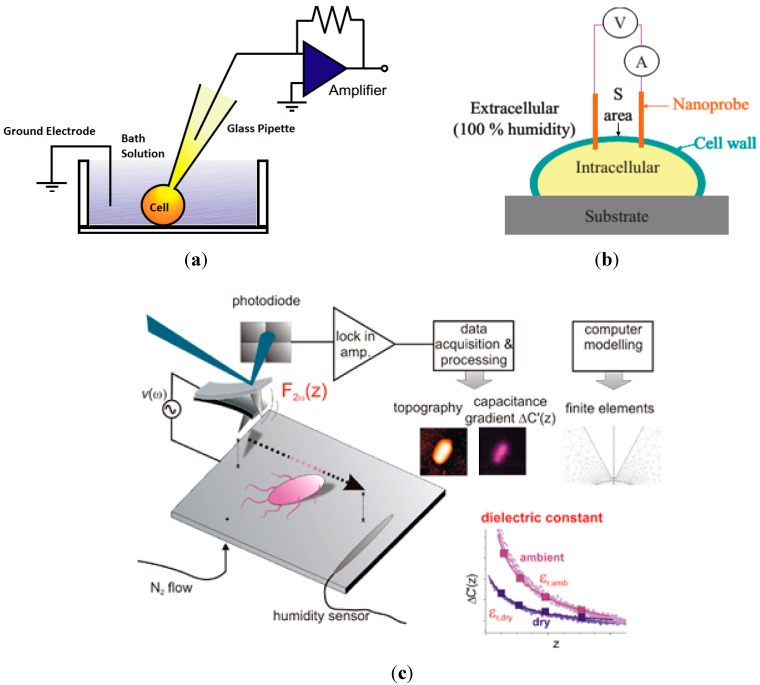
(**a**) Schematic diagram of conventional patch clamp technique; (**b**) Single cell electrical measurement using dual nanoprobes incorporated with ESEM. Reprinted with permission from [37]; (**c**) Schematic of measurement of the effective dielectric constant of a single bacterium using electrostatic force microscopy. Reprinted with permission from [39].

The patch clamp technique is unique in enabling high-resolution recording of the ionic currents flowing through a cell’s plasma membrane. Since the introduction of the patch-clamp technique by Neher and Sakmann in 1976, patch-clamp was adopted by researchers in cellular and molecular biology research areas for studying and providing valuable information of biological cell electrical properties [26,27]. The patch-clamp technique is also capable to analyze ionic currents in the cell membrane under conditions of complete control over transmembrane voltage and ionic gradients. Figure 1a illustrates the basic principle of patch clamp technique. A glass micropipette is used as a probe to suck a cell membrane into a micropipette to form a high electrical resistance or also called as giga-seals (e.g., normally between 10 and 100 GΩ [28]. Thus, the ion current that flows through the pipette (containing an electrode) is measured through an amplifier. Patch clamp can be operated in two modes, which are voltage and current mode. Voltage mode is used to measure voltage specific activity of an ionic channel, while current mode is used to measure the potential change in membrane when a current pulse has been injected into the cell [29]. Furthermore, the patch clamp technique has five basic measurement configurations such as cell-attached patch (CAP), whole-cell (WC), inside-out patch (IOP), outside-out patch (OOP) and permeabilized-patch WC-configuration (ppWC) [30,31]. More detail on the working principle of the patch clamp technique has been described [32,33].

The work of Hamill *et al.* sparked an approach for obtaining information about the characteristics and distribution of ion channels in living cells [28]. They used frog muscle fibres and rat myoballs as cell samples to detail several variants of this technique to create complete electrical isolation of the patched membrane for a variety of cells. This whole cell configuration is the most often utilized mode of the patch clamp technique. Zhang *et al.* combined the whole-cell patch clamp with fluorescence ratio imaging for measuring the electric properties of a cell membrane [34]. Fluorescence dye was used to monitor the transmembrane potential change of the cell in the long term without seriously perturbing the intracellular milieu. Both techniques combined have been successfully used to distinguish between differentiated and undifferentiated N1E-115 neuroblastoma cells according to the values of the resting potentials.

The conventional patch clamp technique has several disadvantages. First, the patch clamp technique is time consuming process [29,35]. The entire dish of cells needs to be replaced after the extracellular fluid has been manipulated, before continue the recording. Second, the quality of the cell and suspension must remain in good condition for channel expression to be homogenous [29]. Third, an experienced operator is required to move the glass pipette over the single cell for measuring current and voltage changes across the membrane through ion channels without damaging the whole cell. Other issues arise such as recoding quality and temperature control. Nevertheless, the patch clamp technique offers high sensitivity (pA resolution) and allows low noise measurement of the currents passing through the low conductance (pS) ion channels [25]. The evolution of upgraded modifications of the patch clamp technique can be found elsewhere [36].

### 2.2. Nanoprobe

Nanoprobes could potentially be used to perform single cell’s electrical characterization. The nanoprobe capable to measure direct electrical properties of single cell and quantitatively determine the viability of single cells. M. R. Ahmad *et al.* developed a dual nanoprobe integrated with nanomanipulator units inside environmental scanning electron microscope (ESEM) to perform electrical probing on single cells for novel single cell viability detection [37]. Figure 1b illustrated the working principle of dual nanoprobe for single cell electrical measurement. Based on Ohm’s law, current flow passing through the intracellular area of the cell was measured when a dual nanoprobe penetrated the intracellular area. ESEM was used for high resolution observation while preserving the cell’s native state even when the cell is moving out of its buffer [38]. This technique successfully differentiated the live and dead cells of W303 wild yeast cells based on the electrical properties of the cell [37]. Recently, electrostatic force microscopy (EFM) was utilized to quantify the electric polarization response of single bacterial cells with high accuracy and reproducibility [39]. They demonstrated effective dielectric constants obtained from the different bacterial types (*Salmonella typhimurium*, *Escherichia coli*, *Lactobacillus sakei* and *Listeria innocua*), which were well correlated with the hydration state of bacteria. Figure 1c illustrated the working procedure of effective dielectric constant measurement using EFM. The electric polarization force between a bacterium and a nanometric-conducting tip mounted on a force-sensing cantilever was measured at different positions. Topographic images were used to obtain the geometry of the bacterium and finite element numerical simulations of a homogeneous bacterium were utilized to measure the effective dielectric constants of the cell [39,40]. A simple single cell electrical model was used in order to measure electrical properties of yeast cells. However, this technique requires a skilled operator to perform the measurement and is time consuming. The device is bulky system, which can only be performed in a restricted area, e.g., clean room [40].

## 3. Microfluidics Platforms

An advance in microfabrication technique, such as soft lithography, creates new opportunities for producing structures at micrometer scale inexpensively and rapidly [41]. For this reason, we have witnessed rapid development of microfluidics system for more than a decade ago for biology and medical research [1,14,42]. Microfluidics systems are a science and technology of manipulating fluids at the submillimetre length scale in the microscale fluidic channel. Microfluidics recognized as micro total analysis systems (µTASs) [43] or lab-on-a-chip (LoC) technologies have attracted attention because of the potential to improve diagnostics and biology research. Microfluidic systems have shown a potential to become widely adopted in modern clinical diagnosis and biology research (e.g., DNA analysis [44] and cell analysis [45]) because they are reproducible, have low power consumption, less sample and reagent consumption, are economical, amenable to modifications and can be integrated with other technologies [46,47]. The ability of microfluidics system to perform early cancer detection and address some problems in cellular analysis, make them suitable to replace the classical technique in single cell electrical analysis. Several microfluidic systems have been developed for single cell electrical properties analysis, such as electrorotation, impedance flow cytometry and microelectrical impedance spectroscopy (µEIS).

### 3.1. Electrorotation

A cell shows a rotated ability when it is placed into a rotating electric field within a medium with a non-uniform electric field. Analysis of these phenomena called an electrorotation (ROT), is commonly used for measuring the dielectric properties of cells without invasion. ROT measurement theory is based on rotational speed of cells/particles when the cell and the suspending medium have different electric polarizability, by referring to the frequency of a rotational electric field. This electric field is generated by quadrupole (arranged in a crisscross pattern) electrodes and each electrode is connected to an AC signal with a 90° phase difference from each other.

**Figure 2:**
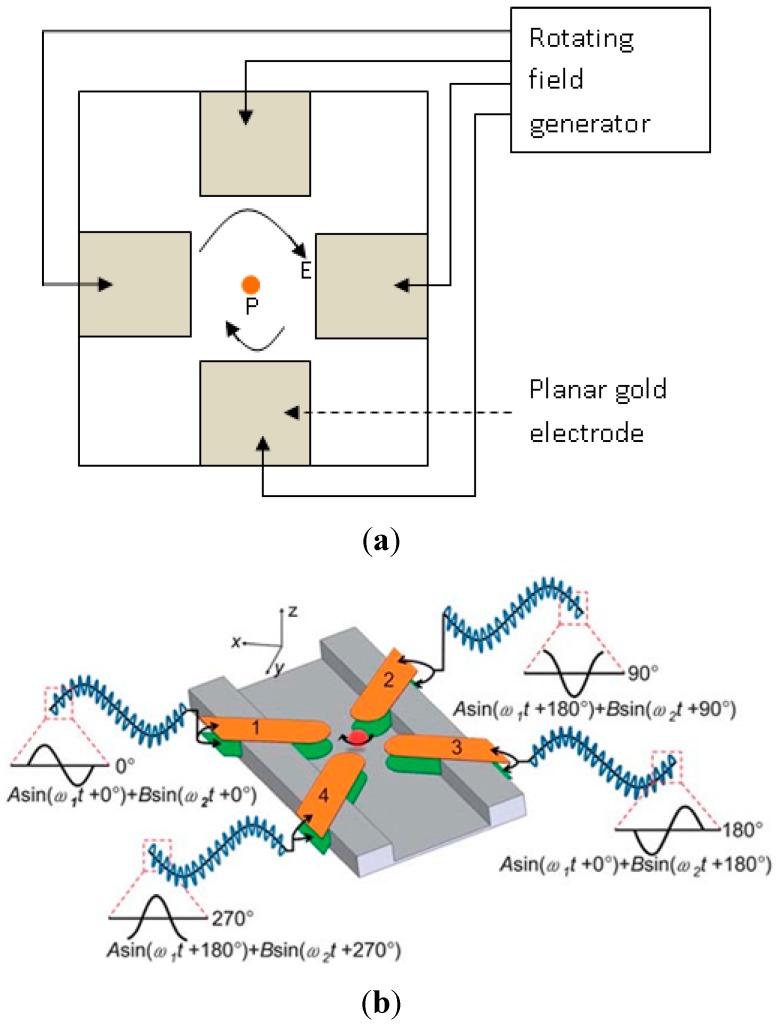
(**a**) An illustration of the working principle of electrorotation to analyse single cells; (**b**) The electrorotation (ROT)-microchip incorporated with the 3D octode. nQDEP (negative quadrupole dielectrophoresis) signal, Asin (ω_1_*t* + 0°) and Asin (ω_1_*t* + 180°) are used for a single cell trapping, while the ROT signals, Bsin(ω_2_*t* + 0°), Bsin (ω_2_*t* + 90°), Bsin (ω_2_*t* + 180°) and Bsin (ω_2_*t* + 270°) are used to simultaneously generate torque. Reprinted with permission from [56].

The quadrupole electrodes connected to sine wave was a famous design in ROT technique [48,49,50]. Figure 2a shows a working principle of ROT, four electrodes were energized by sinusoidal signal generator created rotating electrical field, E. Laser tweezers were used to drag a single cell to the center of a four-electrode chamber, then a single cell, P will rotate in either the same direction (co-field) or in the opposite direction (anti-field) to the rotating field [51]. The direction was taken by the cell depending to the dielectric properties of the cell and suspending medium along with the frequency of the electric field. The dielectric properties of a single cell can then be extracted by utilizing Maxwell’s mixture theory, to associate the complex permittivity of the suspension to the complex permittivity of the cell [49]. More detail on theory and working principle of electrorotation can be found in other articles [52,53,54] and a book [55].

In the ROT technique, the amplitude of the electric field remains unchanged because the cells are only rotated at a certain position in an electric field [57]. Therefore, it is suitable for fitting the rotation spectra at frequency range from 1 kHz to around 200 MHz to determine the intrinsic electrical properties of single cells such as cytoplasm conductivity, cytoplasm permittivity and specific membrane capacitance [48,58,59]. Electrorotation spectra are referred to cellular rotation rate *versus* frequency of the applied field. Jun Yang *et al.* [48] used frequency range between 1 kHz and 120 MHz, to fitting the rotation spectra in order to extract dielectric properties (membrane capacitance) of four main leukocyte subpopulations, *i.e.*, T- and B-lymphocytes, monocytes, and granulocytes. From this experiment, ROT was capable to characterize the dielectric properties of cell subpopulations within a cell mixture. In addition, ROT was utilized to determine the cell viability at real time assessment [51,60,61]. C. Dalton demonstrated that electrorotation technique can be used to determine the viability of two intestinal parasites, *i.e.*, *Giardia Intestinalis* and *Cyclospora Cayetanensis* [51]. An ellipsoidal two-shell model [58] was utilized to analyse the data and estimate the electrical parameter value.

Recently, the concept of negative quadrupole dielectrophoresis (nQDEP) and ROT signals superposed on each other in electrorotation technique was reported (Figure 2b). An accurate ROT spectrum was measured without any other disturbances because repulsive force by the nQDEP signal is stronger [56]. Specific membrane capacitance and cytoplasm conductivity of human leukocyte subpopulations (T-lymphocytes, B-lymphocytes, granulocytes, and monocytes) and metastatic cancer cell lines (SkBr3 and A549) were well achieved. Although the electrorotation technique is powerful tool, capable of extracting the electrical properties of the cell, such as cytoplasm conductivity and membrane permittivity, ROT technique has several drawbacks. Time consumption is a major factor for why the ROT technique has been unable to enter the modern clinical disease diagnosis as an analysis tool. G. De Gasperis *et al.* and M. Cristofanilli *et al.*, utilized ROT technique to analyze single cells and it took approximately 30 min to test a single cell [62,63]. These reports indicate that electrorotaion is a slow technique. Electrorotation also requires a skilled operator to position a single cell in the middle of a rotating electric field and also count the number of revolutions made by particles [64]. Nevertheless, electrorotation is a noninvasive technique which allows it to be used in sequential investigations. ROT also operates at a single-organism level and does not require extensive cell preparations [59]. Table 1 shows a summary of a microfluidic device using electrorotation technique for single cell electrical characterization.

**Table 1:** Microfluidic electrorotation device for single cell electrical analysis.

Authors	Techniques	Experimental Samples	Frequency	Dielectric Parameter	Summary	Reference
X.B. Wang *et al.* (1994)	Four electrode in phase quadrature	DS19	10 kHz–100 Mhz	Specific membrane capacitance	Specific membrane capacitance was determined by the complexity of surface features.	[65]
				DS19 (1.82 ± 0.24 µF/cm^2^)		
				DS19-HMBA (1.6 ± 0.25 µF/cm^2^)		
F.F. Becker *et al.* (1995)	Four electrode in phase quadrature	MDA231, T lymphocytes and Erythrocytes	1 kHz–1 GHz	Specific membrane capacitance	Specific membrane capacitance, cytoplasm conductivity, and cytoplasm permittivity values were reported.	[66]
				MDA231 (26 ± 4.2 mF/m^2^)		
				T lymphocytes (11 ± 1.1 mF/m^2^)		
				Erythrocytes (9 ± 0.80 mF/m^2^)		
R. Hoizel (1997)	Four electrode in phase quadrature	Yeast cells	100 Hz–1.6 GHz	Membrane capacitance yeast (0.76 µF/cm^2^)	Specific capacitance of plasma membrane, periplasmic space and outer wall region values were reported.	[67]
J. Yang *et al.* (1999)	Four electrode in phase quadrature	Leukocyte (WBCs)	10 kHz–120 Mhz	Specific membrane capacitance	Four main leukocyte subpopulations were discriminate based on their electrical properties.	[48]
				T-lymphocytes (10.5 ± 3.1 mF/m^2^)		
				B-lymphocytes (12.6 ± 3.5 mF/m^2^)		
				Monocytes (15.3 ± 4.3 mF/m^2^)		
				Granulocytes (11.0 ± 3.2 mF/m^2^)		
C. Dalton (2001)	Four electrode in phase quadrature	Giardia intestinalis and Cyclospora cayetanensis	20–400 kHz	Membrane conductivity	Viable and nonviable Giardia intestinalis was differentiated based on dielectric parameter value.	[51]
				Giardia intestinalis		
				2 ± 0.81 µS·m^−1^ (viable) &		
				10 ± 0.2 µS·m^−1^ (nonviable)		
M. Cristofanilli *et al.* (2002)	Four electrode in phase quadrature	MCF/neo,MCF/HER2-11 and MCF/HER2-18	10 kHz–100 MHz	Specific membrane capacitance	Specific membrane capacitance of breast cancer cell lines was reported.	[63]
				MCF/neo (2.09027 µF/cm^2^)		
				MCF/HER2-11 (1.70481 µF/cm^2^) MCF/HER2-18 (2.5684 µF/cm^2^)		
S. Han (2013)	Four electrode in phase quadrature	Leukocyte (WBCs), SkBr3 and A549	10 kHz–10 MHz	Specific membrane capacitance	Specific membrane capacitance and cytoplasm conductivity of WBCs and cancer cells was determined using a single-shell dielectric model.	[56]
				T lymphocytes (7.01 ± 0.91 mF/m^2^)		
				B lymphocytes (10.33 ± 1.6 mF/m^2^)		
				Granulocytes (9.14 ± 1.06 mF/m^2^)		
				Monocytes (11.77 ± 2.12 mF/m^2^)		
				SkBr3 (14.83 ± 1.74 mF/m^2^)		
				A549 (16.95 ± 2.93 mF/m^2^)		

### 3.2. Impedance Flow Cytometry

**Figure 3:**
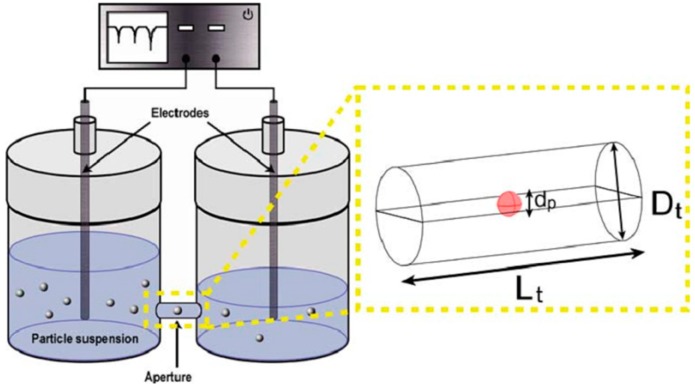
Schematic diagram of the Coulter counter working principle. Reprinted with permission from [72].

Flow cytometry is a fundamental and powerful analytical tool in cell biology and cellular disease diagnosis for many years. Flow cytometry has an ability to address some problems in single cell analysis such as identifiying, counting and sorting cells [68,69]. Based on laser-induced fluorescence detection in flow cytometry for single-cell studies within cell populations of relatively large sizes [70], flow cytometry creates an ideal scenario to analyze single cell electrical properties from a cell population. Coulter [71] developed the first flow cytometry tool having capability to measure the electrical properties of single particles, which is known as the microfluidic Coulter counter. A Coulter counter measures the changing of DC resistance between two electrically isolated fluid-filled chambers when microparticles act as an insulating layer at DC pass through a small connecting orifice. Figure 3 illustrates the working principle of the Coulter counter, where two large electrodes are placed on connecting chamber. When a particles or biological cells flow through a sensing aperture which has current flow, it will displace the conductive fluid and alters the resistance. The current flow was decreased as a particle passes through and for this reason, individual cells can be counted and sized [72]. The Coulter counter is limited to counting cells and classifying cell types based on size due to challenging of selecting electrode design and channel geometry [73]. The Microfluidic Coulter counter is incapable of characterizing electrical properties of cell. In order to determine the electrical properties of cells, Sohn *et al.* [74] developed flow cytometry based on capacitance principle to measure the DNA content of fixed eukaryotic cells. Electrical properties of individual cells were referred to distinct peaks measured by a capacitance bridge at 1 kHz frequency.

**Figure 4:**
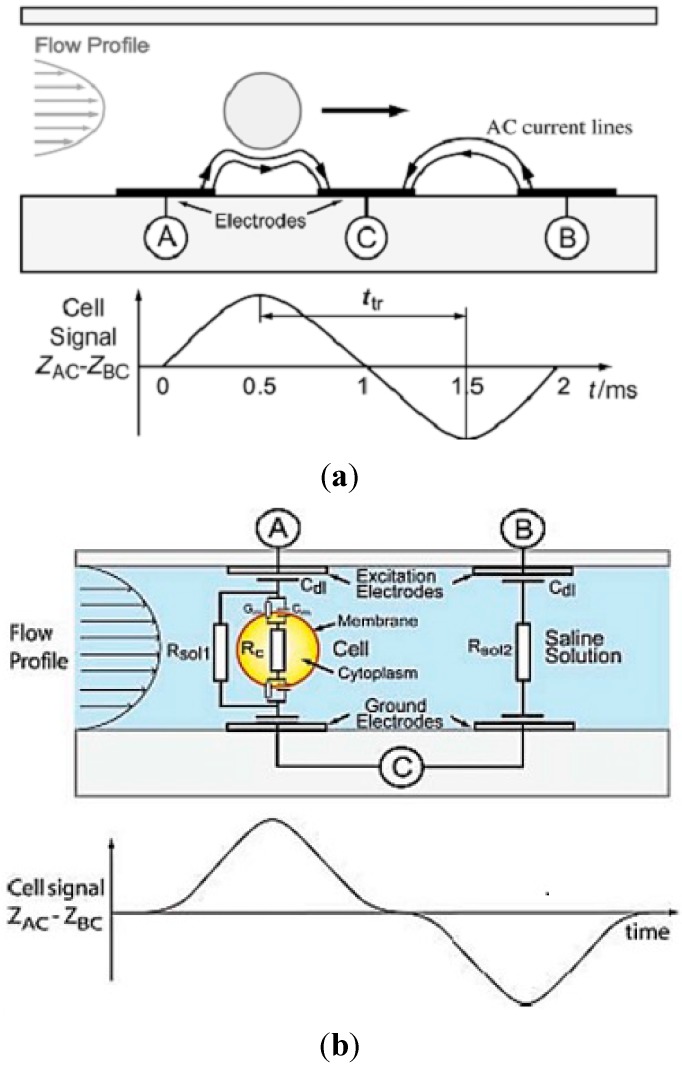

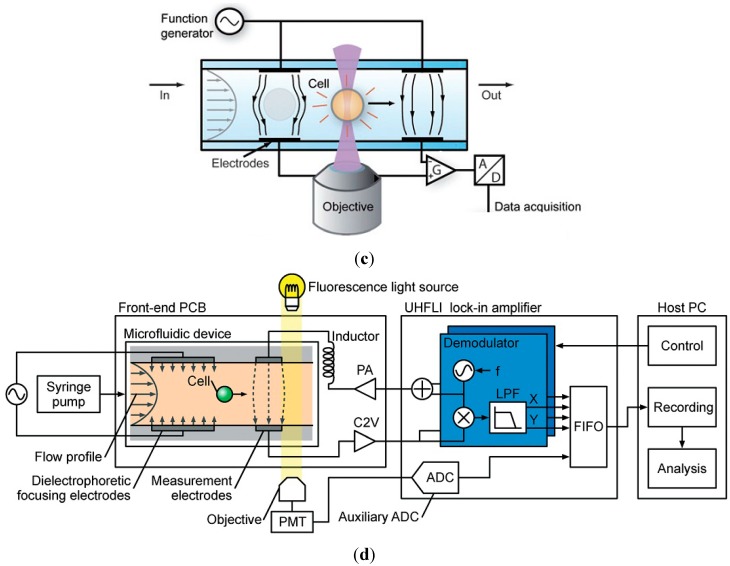
(**a**) Illustration of a particle flowing over three electrodes inside a microfluidic channel, and a typical impedance signal for a single particle. Reprinted with permission from [75]; (**b**) A single cell flowing over one pair of electrode and second pair used as reference is shown. Reprinted with permission from [76]; (**c**) Schematic diagram of the micro impedance cytometer system, including the confocal-optical detection. Reprinted with permission from [81]; (**d**) Schematic of the complete microfluidic cytometer. The lock-in amplifier drives the series resonance circuit, formed by the discrete inductor and the impedance between the measurement electrodes, with an alternating current (AC) signal at a frequency close to resonance. Reprinted with permission from [82].

Gawad *et al.* [75] developed a significant device in single cell impedance technology, which is known as the impedance flow cytometry (IPC). This device used coplanar electrodes to measure clear differentiation of beads and also erythrocytes and ghost cells (ghosts are RBCs that have been lysed in hypotonic buffer, leaving behind a membrane sack filled with ionic solution). As shown in Figure 4a, three microelectrodes were fabricated on the bottom of a microfluidic channel. An AC voltage was supplied to energise the electrodes for generating a non-uniform electric field within the channel. The impedance value within channel was changed, when a single cell was flowing through the detection area. This impedance value was used to characterize the electrical properties of single cell. However, this electrode configuration may affect impedance measurement when single cell was at variation position. To address this issue, K. Cheung *et al.* [76] designed parallel facing electrodes in a microfluidic channel (Figure 4b). One pair of parallel electrodes was used to detect cells and measure electric current fluctuation, whereas the other one was acted as a reference. Then, the difference between the two signals was measured. The device has the ability to measure electrical properties of normal RBCs and glutaraldehyde-fixed RBCs. More details for the derivation of the electric field distribution for two different electrode configurations, based on Schwarz–Christoffel Mapping (SCM) have been described [77]. In addition, a similar system (parallel facing electrodes) was used by Kampmann *et al.* [78] to monitor frequency effect during conducted measurement processes. The result showed that the cell can be accurately sized at around 500 kHz, where low frequency behaviour is dominated by the electrical double layer (EDL). Meanwhile, at intermediate frequencies behaviour is dominated by the membrane capacitance and at high frequencies, the cell cytoplasm becomes important. High frequency (8.7 Mhz) measurements were used to detect infection of RBCs with the parasite Babesia bovis based on the changes in the electrical properties of the cell cytoplasm [79]. Recently, an impedance flow cytometry that covers frequency range from DC up to 500 Mhz was developed by Niels Haandbæk *et al.* [80]. The device has a capability of dielectric characterization of subcellular components of yeast cells, such as vacuoles and cell nuclei, and can be used for discriminating wild-type yeast from a mutant.

Holmes *et al.* [81] demonstrated measurement and differential of single cells at a high speed level by using microfluidic flow cytometry with an attached fluorescence measurement unit (Figure 4c). The device accurately identified T-lymphocytes, monocytes and neutrophils of WBC and a full three-part differential count of whole human blood was achieved. Despite single cells being measured, the data represented the average of the population and was not accurately measured on an individual cell. In addition, similar research groups developed an integrated microfluidic impedance flow cytometry system with haemoglobin concentration measurement unit [83] and RBC lysis [84]. In order to increase the signal-to-noise ratio relative to a single-phase, unfocused stream, while to avoid large shear forces on cells, Mikael Evander [85] developed a microfluidic impedance cytometer that utilizes dielectrophoretic focusing technique. This technique was used to center cells in a fluid stream, thus forms the core of a two-phase flow. Then, this flow will pass between electrodes for analysis of cells at various frequencies from range 280 kHz to 4 MHz. As a result, this technique is able to distinguish between red blood cells and platelets and between resting and activated platelets.

A label-free cell cytometry based on electrophysiological response to stimulus was reported [86]. This method recorded a cell’s functionality rather than its expression profile or physical characteristics. In order to distinguish different cells types, they used nature electrically excitable cells that are activated by sufficient transmembrane electric fields. During this activation, the extracellular field potential (FP) signal from cells was produced and detected by electrode inside microchannel. Human induced pluripotent stem cell-derived cardiomyocyte (iPSC-CM) clusters from undifferentiated iPSC clusters were differentiated by using these signals. A contactless measurement method to perform single cell impedance cytometry using a disposable biochip integrated with a printed circuit board that has reusable electrodes was reported [87]. The device can detect and measure impedance of biological cells in a real biological sample (e.g., whole blood (sheep)) and also significantly reduces the manufacturing costs. Recently, a microfluidic impedance cytometer, incorporated with an electrical resonator was reported. This device is high sensitive and capable to measure at high frequencies. Figure 4d showed microfluidic system integrated with a resonant circuit which consists of a discrete inductor in series with the impedance between the measurement electrodes [82]. The cells detection principle is based on the resonance- enhanced phase shift of the measurement current induced by cells or particles passing through the microfluidic channel. Discrimination based on the differences in dielectric properties of *E. coli* and B. subtilis was well achieved. T. Sun *et al.* extends impedance measurements from one dimension to two or three dimensions by utilized electrical impedance tomography (EIT) [88]. A circular 16-electrode array with equal spacing was fabricated and images of Physarum polycephalum were reconstructed by measuring the voltages across sequential electrode-pair combinations. Human fibroblast cells were used to differentiate between an environment of growth medium with and without cells using EIT [89]. Table 2 shows a summary of microfluidic impedance flow cyometry techniques for single cell electrical characterization.

**Table 2:** Microfluidic impedance flow cytometry device for single cell electrical analysis.

Authors	Techniques	Experimental Samples	Frequency	Summary	Reference
K. Cheung *et al.* (2005)	Parallel facing electrodes	RBCs, ghost RBCs and fixed RBCs	602 kHz and 10 MHz	Controlled RBCs, ghost RBCs and fixed RBCs were distinguished using impedance opacity.	[76]
G. Benazzi *et al.* (2007)	Coplanar electrodes	Algae	327 kHz and 6.03 MHz	Three populations of algae were distinguished on the basis of impedance measurement.	[90]
C. Kuttel *et al.* (2007)	Coplanar electrodes	Babesia bovis infected RBCs	8.7 MHz	The real part and imaginary part of the impedance signal were used for cell type classification.	[79]
G. Schade-Kampmann *et al.* (2008)	Parallel facing electrodes	Jurkat cell, yeast cell and 3T3-L1	624 kHz and 1–15 MHz	Various cell lines, human monocytes and *in vitro*-differentiated dendritic cells and macrophages, viable and apoptotic Jurkat cells were discriminated. Yeast cell growth was also monitored using impedance measurement.	[78]
Y. Katsumoto *et al.* (2008)	Parallel facing electrodes	rabbit erythrocytes and human erythrocytes	10 kHz–100 MHz	Specific membrane capacitance and cytoplasm conductivity values were determined from their dielectric dispersion using new numerical method based on rigorous electric-field simulation combined with three-dimensional modeling of an erythrocyte.	[91]
D. Holmes *et al.* (2009)	Parallel facing electrodes	WBCs	573 kHz and 1.7 MHz	Microfluidic impedance flow cytometry was incorporated with fluorescence detection.	[81]
K.C. Cheung *et al.* (2010)	Parallel facing electrodes	Macrophage, MCF-7, RN22, blood cells and yeast	0.5–15 MHz	Macrophage differentiation, cell viability, blood cells, and RN22 with altered membrane potential and intercellular calcium concentration were distinguished.	[92]
C. Bernabini (2011)	Parallel facing electrodes + hydrodynamic focus	*E. coli* and 1 & 2 µm beads	503 kHz	A focusing technique mitigated the clogging issue and increased sensitivity.	[93]
J. Chen *et al.* (2011)	Constriction channel	MC-3T3	100 Hz–1 MHz	Specific membrane capacitance and cytoplasm conductivity values were determined using a simple equivalent circuit models.	[94]
X.J. Han (2012)	Parallel facing electrodes	RBCs and WBCs	573 kHz–1.7 MHz	The functions of blood dilution, RBCs lysis, and hemoglobin detection were integrated.	[84]
G. Mernier (2012)	Liquid electrodes + DEP focusing	Yeast Cells	500 kHz–15 Mhz	DEP was applied to reduce measurement variations by focusing particles in the middle of the channel.	[95]
Y. Zheng (2013)	Constriction channel + 7 fequencies measurement	AML-2 and HL-60	1–400 kHz	Specific membrane capacitance and cytoplasm conductivity values were determined at speed of 5–10 cell·s^−1^.	[96]
F.B. Myers (2013)	Electrophysiological cytometry	Pluripontent stem cells	N/A	Clusters of undifferentiated human-induced pluripotent stem cells (iPSC) were identified from iPSC-derived cardiomyocyte (iPSC-CM) clusters.	[86]
Haandbæk *et al.* (2014)	Parallel facing electrodes + resonant circuit	*E. coli* and B. subtilis	89.2 and 87.2 MHz	Discrimination based on the differences in dielectric properties of *E. coli* and B. subtilis.	[82]

RBC: red blood cell; WBC: white blood cell.

### 3.3. Micro Electrical Impedance Spectroscopy (µ-EIS)

Micro electrical impedance spectroscopy (µ-EIS) is a technique where dielectric properties in a frequency domain of a cell is measured to characterize and differentiate the various types of cell. Mainly this technique analyzed the current response when a single cell was trapped in a trapping system where an alternating current (AC) was applied across the trapping zone. A trapping system is a major contribution and significant part in µ-EIS device. For this reason, development of a trapping system is very crucial and varieties of the trapping system have been developed, such as hydrodynamic traps, negative pressure traps and DEP traps.

**Figure 5:**
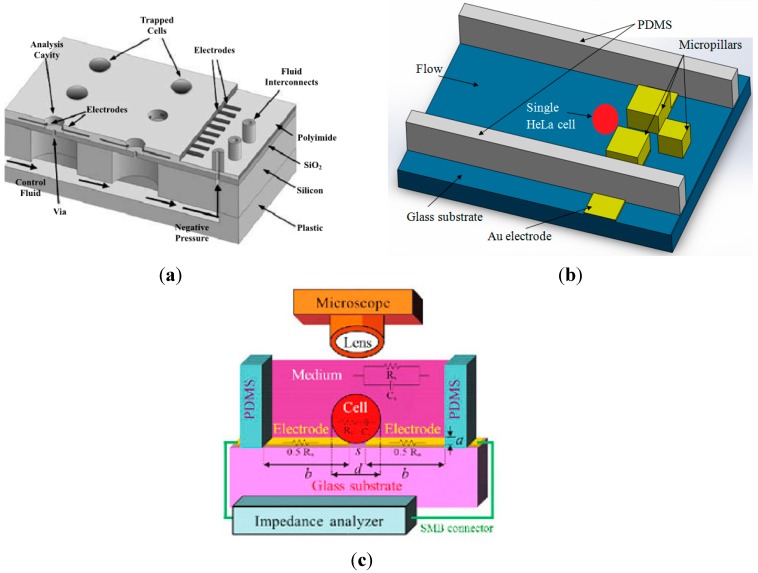
(**a**) Illustrated a micro electrical impedance spectroscopy system using multielectrode configurations within an analysis cavity. Reprinted with permission from [97]; (**b**) Shown 3D schematic of the µ-EIS device incorporated with micropillars structure for capture the single cells; (**c**) Schematic diagram of cell measurement using DEP cell trapping technique. Reprinted with permission from [104].

First development of micro electrical impedance spectroscopy (µ-EIS) was reported in 2006 [97]. They developed microfluidic device which utilized the negative pressure to capture the single cell into the analysis cavity (Figure 5a). This device was used to measure the electrical impedance of human breast cancer cell lines of different pathological stages (MCF-7, MDA-MB-231, and MDA-MB-435) [18]. However this device has a disadvantage to monitor the cell capturing process using a microscope because the contrast difference in the silicon nitride membrane composing the cell traps area and the surroundings. The same group, Cho *et al.* [4] developed an array of horizontal cell traps of an µ-EIS device to overcome the limitation of the previous device. Negative pressure was used to capture single cells and impedance measurement was performed to obtain the electrical impedance spectra of metastatic head and neck cancer (HNC) cell lines. This device also can minimize the leakage current due to the position of cells formed in direct contact between cells and electrodes.

Furthermore, the concept of vertical trapping system in µ-EIS has been used to monitor the dynamic change of single cell electrical properties over a period of time [98,99]. Hydrodynamic trapping system (e.g., micropillars) within a microfluidic channel was developed by Jang *et al.* [21]. Figure 5b showed the micropillars structure inside microfluidic channel and capable to capture physically single cells. A single human cervical epithelioid carcinoma (HeLa) was successfully captured by the micro pillars and its impedance was measured. Mondal *et al.* performed impedance measurement of HeLa cell based on two geometry structures of micropillars trapping system, namely, parallel and elliptical geometry [100]. Malleo *et al.* demonstrated a hydrodynamic trapping device which has a differential electrode arrangement that measures multiple signals from multiple trapping sites. Measurements was performed by recording the current from two electrode pairs, one empty (reference) and one containing HeLa cells [101]. The device continuously monitored the toxin activity at the single cell level.

Recently, the concept of dielectrophoresis (DEP) for trapping system was reported [102]. The non-uniform electric field distribution between the top and bottom electrodes caused the red blood cells (RBCs) to experience positive dielectrophoresis at 80 kHz frequency [103]. As a result, the red blood cells have been trapped inside microwells, thus the impedance of RBCs was measured [102]. Another DEP trapping technique was developed by Tsai *et al.* to capture a single HeLa cell, then impedance measurement was performed [104]. Figure 5c illustrated trapping system using DEP [104]. Despite that microelectrical impedance spectroscopy (µ-EIS) has several advantages such as label free, real time measurement and non invasive, µ-EIS also has some drawbacks. For example, µ-EIS requires theoretical model for data analysis [105] and time consuming (trapping and releasing process take time to be completed) [106]. Table 3 shows a summary of microelectrical impedance spectroscopy technologies for analyzing the single cell’s electrical properties.

**Table 3:** Microelectrical impedance spectoscopy device for single cell electrical analysis.

Authors	Techniques	Experimental Samples	Frequency	Dielectric Parameter	Summary	Reference
A. Han *et al.* (2003)	Vertical hole	MCF-7,MCF-10A, MCF-MB-231 and MDA-MB-435	100 Hz–3 MHz	specific membrane capacitance	Impedance spectra were shown to be significantly different between the normal cell lines and each of the cancer cell lines.	[97]
				MCF-10A (1.94 ± 0.14 µF/cm^2^)		
				MCF-7 (1.86 ± 0.11 µF/cm^2^)		
				MDA-MB-231 (1.63 ± 0.17 µF/cm^2^)		
				MDA-MB-435 (1.57 ± 0.12 µF/cm^2^)		
L.S. Jang *et al.* (2007)	Micropillars	Hela	1 Hz–100 kHz	cell membrane C_c_	A circuit model was developed to obtained and calculate electrical parameters of HeLa cells.	[21]
				2.5 × 10^−12^ F		
				cytoplasm R_c_		
				6 × 10^7^ Ω		
S.B. Cho *et al.* (2007)	Vertical hole	L929	1 Hz–100 MHz	N/A	A culture of L929 cells and the toxicity effect on impedance measurement were monitored on the micro hole. Cell growth and the membrane integrity can monitored without any labelling.	[98]
Y. Cho *et al.* (2009)	Parallel lateral trapping holes	686LN and 686LN-M4e	40 Hz–10 MHz	N/A	The phase part of impedances could be used to differentiate the poorly metastatic cell line from the highly metastatic cell line.	[4]
D. Malleo *et al.* (2010)	Hydraulic trapping	Hela	300 kHz	N/A	Effect of a surfactant and a pore-forming toxin on captured cells was monitored by referring the impedance value of captured cells.	[101]
C.L. Kung *et al.* (2011)	DEP trapping	Hela	1 Hz–100 kHz	N/A	An alternating current electrothermal effect (ACET) and a negative dielectrophoresis (nDEP) force was utilized to trap cells.	[107]
C.M. Kurz *et al.* (2011)	Vertical hole	Arpe-19	1 kHz	N/A	The subtoxic effect of cells was measured by monitoring impedance signals over time.	[99]
Y. Zhao *et al.* (2014)	Constriction channel + impedance measurement	95D and 95D CCNY-KD	1 and 100 kHz	specific membrane capacitance	Specific membrane capacitance and cytoplasm conductivity were determined.	[7]
				95D (1.8–2.0 μF/cm^2^)		
				95D CCNY-KD (1.4–1.6 μF/cm^2^)		
P. Shah *et al.* (2014)	pDEP trapping	CCL-149 (Rat lung epithelial cells)	1 Hz–10 MHz	impedance in absence 1.51 MΩ	Impedance spectrum used to monitoring in absence and in the presence of a single cell in microwell.	[108]
				impedance in the presence of cell 17 MΩ		
S.-B. Huang, *et al.* (2014)	Constriction channel with an incorporated pneumatically driven + impedance measurement	CCL-185	1 and 100 kHz	specific membrane capacitance 2.17 ± 0.58 µF/cm^2^	A pneumatically driven membrane-based active valve was utilized for unblocking cell aggregates at the entrance constriction channel.	[20]
				cytoplasm conductivity 0.74 ± 0.20 S/m		

## 4. Discussion

The rapid development of single cell analysis tools (e.g., biophysical and chemical characterizations) can be seen based on the hundreds of review and technical papers currently published every year [109]. Clearly, the growing interest in this research field demonstrates its practical value from the viewpoint of proof of concept and applications. The traditional platform is a basic foundation to provide the most straight-forward mechanisms to analyze electrical properties of individual cell. However this approach suffers from low throughput, delicate protocol, requires an experienced operator and bulky experimental set-up [110]. For instance, in clinical application, high throughput devices are significantly required to test a large number of cells (e.g., blood) in order to obtain low numbers of meaningful data (e.g., CTC cells) [57]. Nevertheless, traditional platforms have provided fundamental insights to the microfluidic development. The microfuidics device is a promising technique to understand the cellular heterogeneity and overcome the limitation of traditional technique. For that reason, three microfluidics techniques (electrorotation, impedance flow cytometry and microelectrical impedance spectroscopy) have been developed to analyze and characterize the single cell’s electrical properties. Among these techniques, microfluidic impedance flow cytometry (IFC) is a technique used widely in clinical diagnosis because of high throughput during count and differentiation of the WBCs. For example, parallel facing electrodes device achieved ~100 cells·s^−1^ and is capable of testing a large number of cells for obtain statistically meaningful data [81]. Microfluidic impedance flow cytometry has been demonstrated to distinguish various single cells (16 types of cell) based on the electrical properties conditions. Meanwhile, 13 types of cell were distinguished by electrorotation and microelectrical impedance spectroscopy.

Electrical measurements can also be incorporated with a cell sorting unit to collect cells having different physical properties for further biochemical assaying. AC dielectrophoretic (DEP) for sorting live cells from interfering particles of similar sizes by their polarizabilities under continuous flow was reported [111]. DEP forces induced by the AgPDMS electrodes were used to manipulate cells to move toward high or low electric field regions, depending on the relative polarizability between the cells and their suspending medium. Jun Yang *et al.* utilized magnetically activated cell sorting (MACS) for obtaining the subpopulations from human peripheral blood (B-lymphocytes and monocytes), thus performing the single cell electrical properties measurement by electrorotation techniques [48].

Microfluidic devices have demonstrated great potential in realizing electrical measurements on single cells at a higher testing speed and label free approach. Electrical measurements on single cells can be used to indicate possible diseases and it suitable for disease prescreening application. From prescreening processes, future examinations can be done to evaluate the disease condition. Table 4 shows a summary of comparisons between three microfluidic methods. The microfluidic techniques were discussed have some potential applications in biological and medical application [18,44,92,101,112].

**Table 4:** Comparisons between three microfluidic techniques.

Approaches Technique	Advantages	Disadvantages	Applications
Electrorotation	Capable to quantifying a cell’s intrinsic electrical properties	Low throughput and limitation to low conductivity sucrose buffer solution	Monitor parasite; Cell separation
Impedance flow cytometry	High throughput	low specificity	Cell sorting and counting; Cell impedance variations; DNA hybridization detection
Microelectrical impedance spectroscopy	Characterizing ion channel activity	Low throughput and size-independent parameters	Cancerous stage screening; Toxin detection

## 5. Conclusions

The presented review of selected research works on single cell electrical properties provides information on technological development in single cell electrical characterization from traditional approaches to current microfluidic approaches. Microfluidics technology opens a new paradigm in cellular and microbiology research for early disease detection and provides critical information needed by research scientists and clinicians for improved clinical diagnosis and patient outcome. The recent excellent achievements in microfabrication techniques have enabled the rapid development of microfluidic technologies for further practical applications for the benefit of mankind. Furthermore, microfluidic technological progress has provided additional advantages such as reduced complexity of experiment handling, lower voltage on the electrodes, faster heat dissipation, small volume of reagents used, and *in situ* observation of the cell response [113].
